# Supplementary material for: Comparison of uridine and N1-methylpseudouridine mRNA platforms in development of an Andes virus vaccine
Source: Nat Commun. 2024 Jul 30;15:6421. doi: 10.1038/s41467-024-50774-3 (PMC11289437; doi:10.1038/s41467-024-50774-3)
Supplement: Supplementary file 1 — Supplementary Information [file 41467_2024_50774_MOESM1_ESM.pdf]

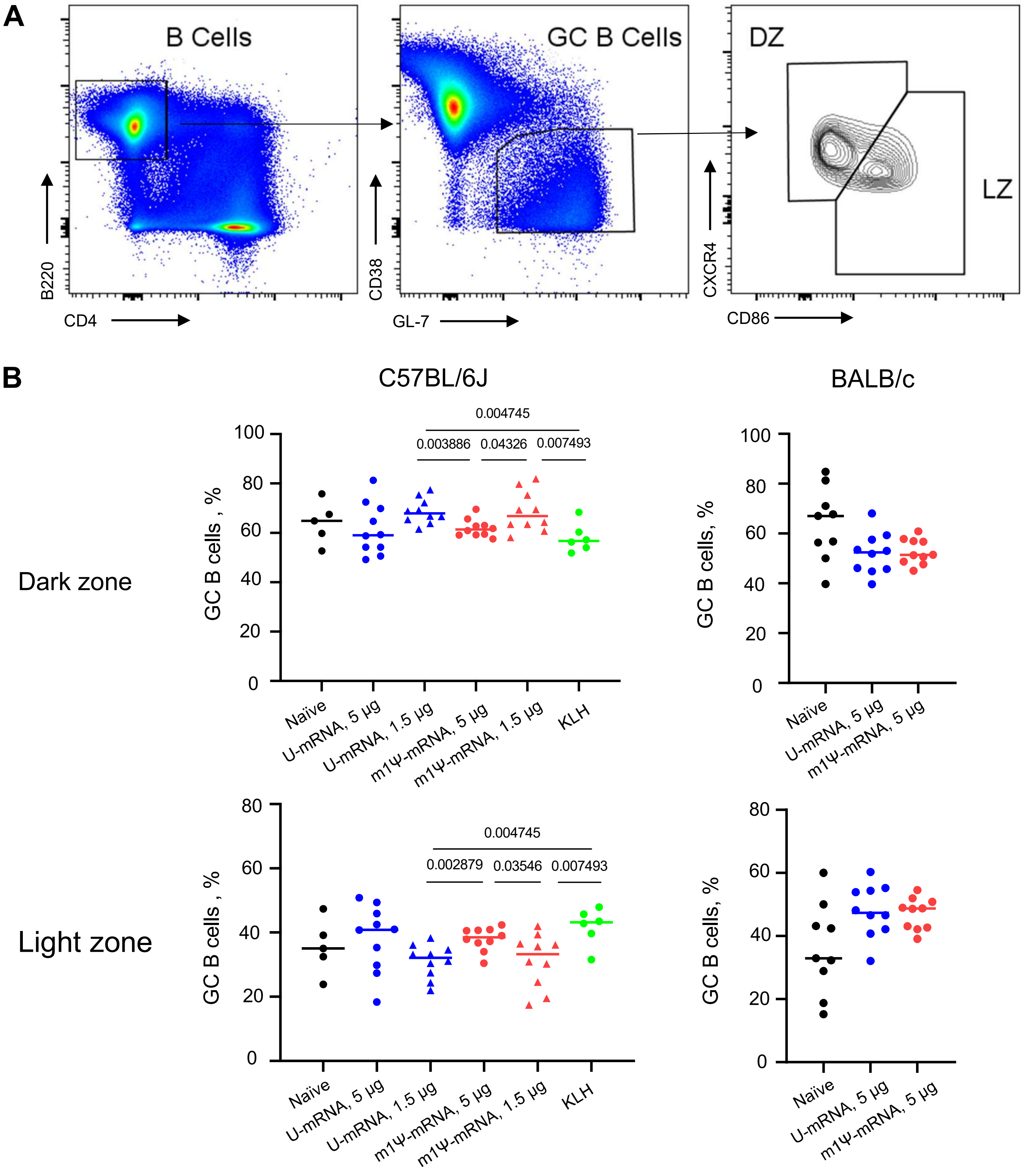

Fig. S1. Analysis of B cells from dark and light zones of germinal center. A. Representative gating strategy to get to DZ (Lymphocytes/Singlet/Live/B220+/CD4-/CD38-/GL7+/CXCR4+/CD86-) and LZ (Lymphocytes/Singlet/Live/B220+/CD4-/CD38-/GL7+/CXCR4-/CD86+) GC B cells. **B.** Percentages of B cells in germinal center DZ and LZ from C57BL/6J and BALB/c mice. (Naïve C57BL/6J = 10 mice, U-mRNA 5 µg C57BL/6J = 10 mice, U-mRNA 1.5 µg C57BL/6J = 10 mice, m1Ψ-mRNA 5 µg C57BL/6J = 10 mice, m1Ψ-mRNA 1.5 µg C57BL/6J = 10 mice, KLH C57BL/6J = 6 mice, Naïve BALB/c = 10 mice, U-mRNA 5 µg BALB/c = 10 mice, m1Ψ-mRNA 5 µg BALB/c = 10 mice**)** Median values, two-tailed unpaired Mann-Whitney test.

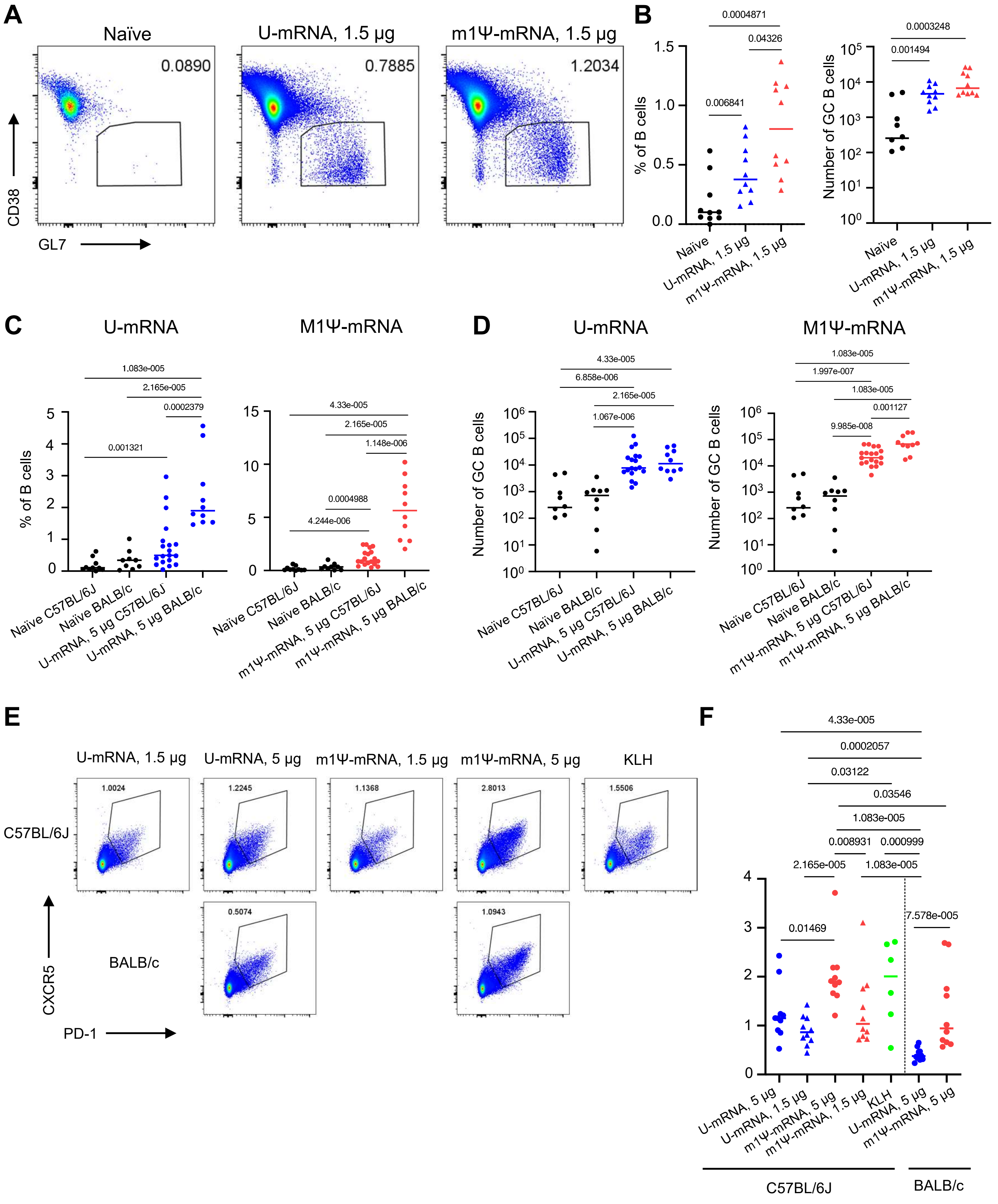
A
Naïve
U-mRNA, 1.5 μg
m1Ψ-mRNA, 1.5 μg
0.0890
0.7885
1.2034
CD38
GL7
B
% of B cells
Number of GC B cells
0.0004871
0.04326
0.006841
0.0003248
0.001494
Naïve
U-mRNA, 1.5 μg
m1Ψ-mRNA, 1.5 μg
C
U-mRNA
M1Ψ-mRNA
% of B cells
1.083e-005
2.165e-005
0.0002379
0.001321
4.33e-005
2.165e-005
1.148e-006
0.0004988
4.244e-006
Naïve C57BL/6J
Naïve BALB/c
U-mRNA, 5 μg C57BL/6J
U-mRNA, 5 μg BALB/c
m1Ψ-mRNA, 5 μg C57BL/6J
m1Ψ-mRNA, 5 μg BALB/c
D
U-mRNA
M1Ψ-mRNA
Number of GC B cells
4.33e-005
6.858e-006
2.165e-005
1.067e-006
1.083e-005
1.997e-007
1.083e-005
0.001127
9.985e-008
E
U-mRNA, 1.5 μg
U-mRNA, 5 μg
m1Ψ-mRNA, 1.5 μg
m1Ψ-mRNA, 5 μg
KLH
C57BL/6J
BALB/c
1.0024
1.2245
1.1368
2.8013
1.5506
0.5074
1.0943
CXCR5
PD-1
F
4.33e-005
0.0002057
0.03122
0.03546
1.083e-005
0.008931
0.000999
2.165e-005
1.083e-005
7.578e-005
0.01469
U-mRNA, 5 μg
U-mRNA, 1.5 μg
m1Ψ-mRNA, 5 μg
m1Ψ-mRNA, 1.5 μg
KLH
U-mRNA, 5 μg
m1Ψ-mRNA, 5 μg
C57BL/6J
BALB/c

Fig. S2. Analysis of germinal center and Tfh populations in response to strain and dose. A. Representative flow cytometry plots for germinal centers from low dose C57BL/6J recipients. **B.** Quantification of GC frequency (left) and total number of GC B cells in log scale from low dose C57BL/6J recipients (right). (Naïve = 10 mice, U-mRNA 1.5 µg = 10 mice, m1Ψ-mRNA 1.5 µg = 10 mice) **C.** Quantification of GC frequency in dose-matched C57BL/6J and BALB/c recipients of either the U-mRNA (left) or m1Ψ-mRNA (right) vaccine. Data set the same as with Fig 2. (Naïve C57BL/6J = 10 mice, Naïve BALB/c = 10 mice, U-mRNA 5 µg C57BL/6J = 18 mice, U-mRNA 5 µg BALB/c = 10 mice, m1Ψ-mRNA 5 µg C57BL/6 = 19 mice, m1Ψ-mRNA 5 µg BALB/c = 10 mice) **D.** Quantification of total number of GC B cells in log scale from both C57BL/6J and BALB/c recipients of either vaccine. Data set is the same as Fig 2. **E.** Representative flow cytometry plots of Tfh (Lymphocytes/Singlet/Live/B220-/CD4+/FoxP3-/CXCR5+/PD-1+) from low and high dose treated C57BL/6J or high-dose treated BALB/c mice. **F.** Quantification of Tfh frequency as percent of total CD4 population. (U-mRNA 5 µg C57BL/6J = 10 mice, U-mRNA 1.5 µg C57BL/6J = 10 mice, m1Ψ-mRNA 5 µg C57BL/6J = 10 mice, m1Ψ-mRNA 1.5 µg C57BL/6J = 10 mice, KLH C57BL/6J = 6 mice, Naïve BALB/c = 10 mice, U-mRNA 5 µg BALB/c = 10 mice, m1Ψ-mRNA 5 µg BALB/c = 10 mice) Two-tailed unpaired Mann-Whitney test.

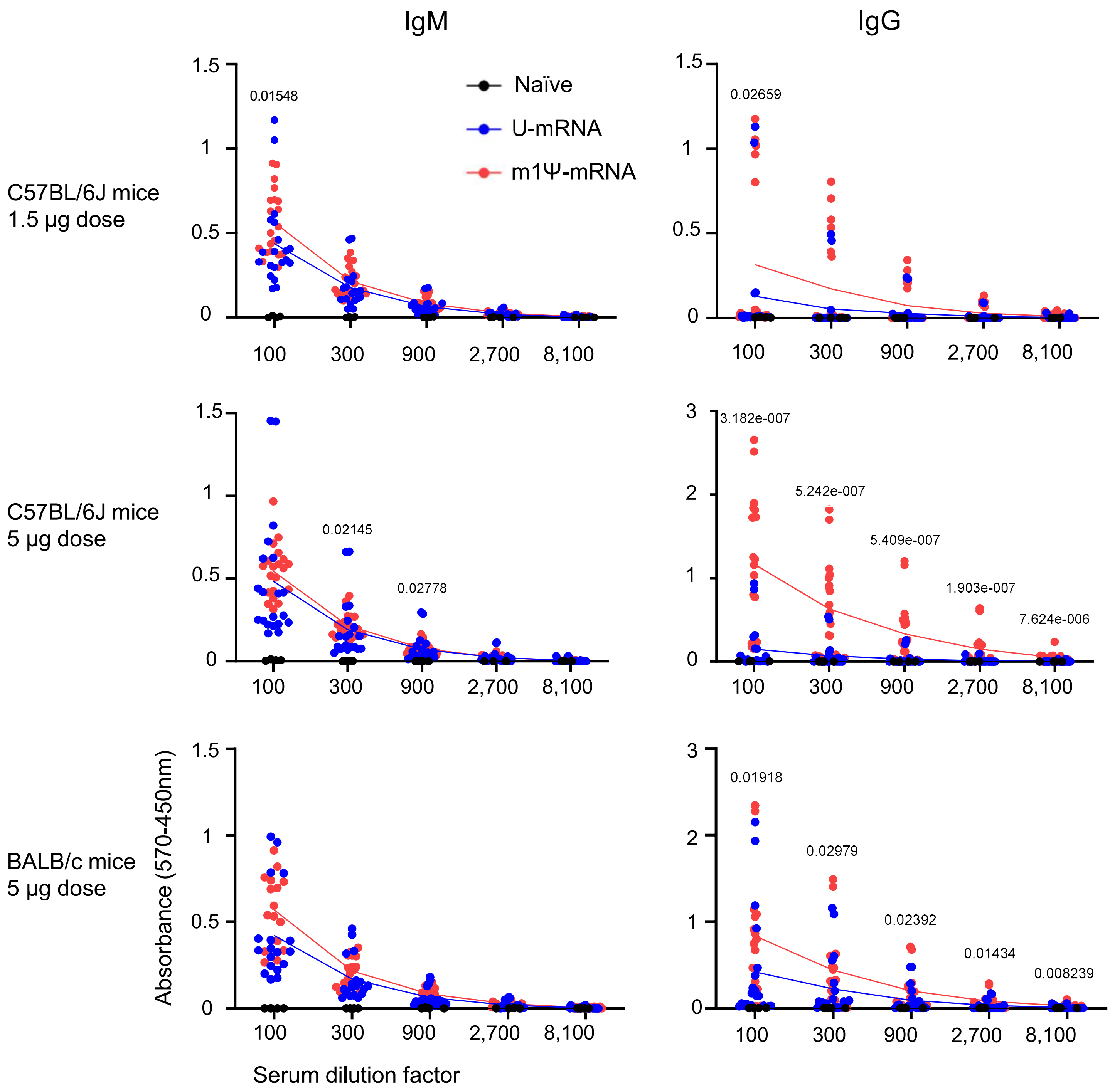

Fig. S3. ANDV GnGc-binding antibody responses determined by ELISA in sera of immunized C57BL/6J and BALB/c mice. Day 12 after immunization with the indicated doses of the vaccines. Each data point represents the mean value of technical duplicates of sera from immunized and naïve mice from two identical experiments (Naïve C57BL/6J = 2 mice, , U-mRNA 1.5 µg C57BL/6J = 10 mice, m1Ψ-mRNA 1.5 µg C57BL/6J = 10 mice, U-mRNA 5 µg C57BL/6J = 10 mice, m1Ψ-mRNA 5 µg C57BL/6J = 10 mice, Naïve BALB/c = 2 mice, U-mRNA 5 µg BALB/c = 10 mice, m1Ψ-mRNA 5 µg BALB/c = 8 mice). The lines connect mean values for every dilution. Two-tailed unpaired Mann-Whitney test.

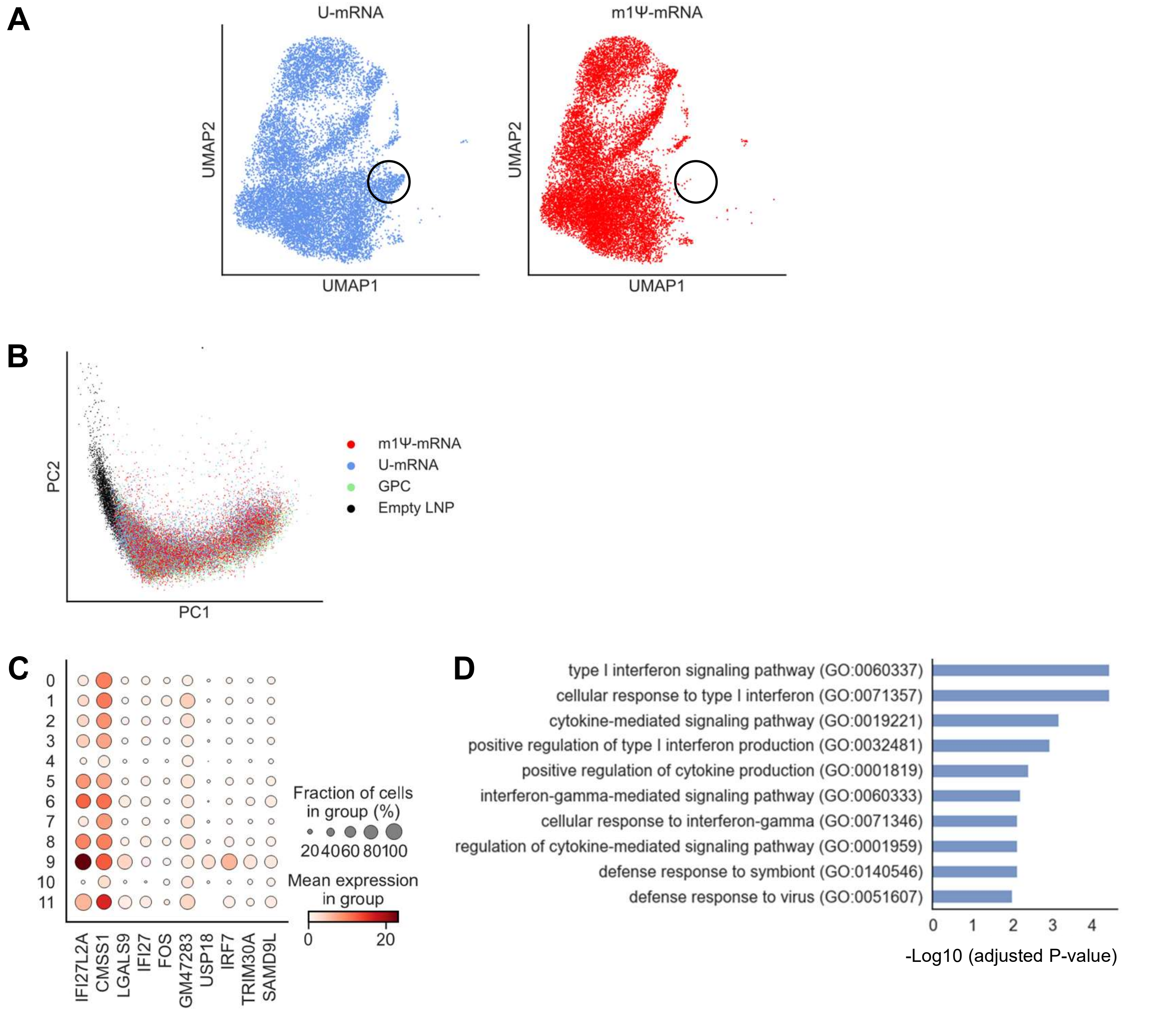

Fig. S4. Cluster 9 of B cells isolated from lymph nodes of mice immunized with U-mRNA vaccine demonstrates upregulation of genes involved in the innate immune response. Data from the study shown in Fig. 3; n = 4 mice per group. **A.** Comparison of single-cell RNA-seq data from lymph nodes isolated from immunized mice: U-mRNA (left) and m1Ψ-mRNA (right). Cluster 9 is circled. **B.** Principal component analysis of B cells from single-cell RNA-seq. **C.** Mean expression of top 10 differentially expressed genes for mice immunized with U-mRNA across clusters. **D.** Top 10 significantly upregulated terms from the Gene Ontology Biological Processes database using the differentially expressed genes for Cluster 9.

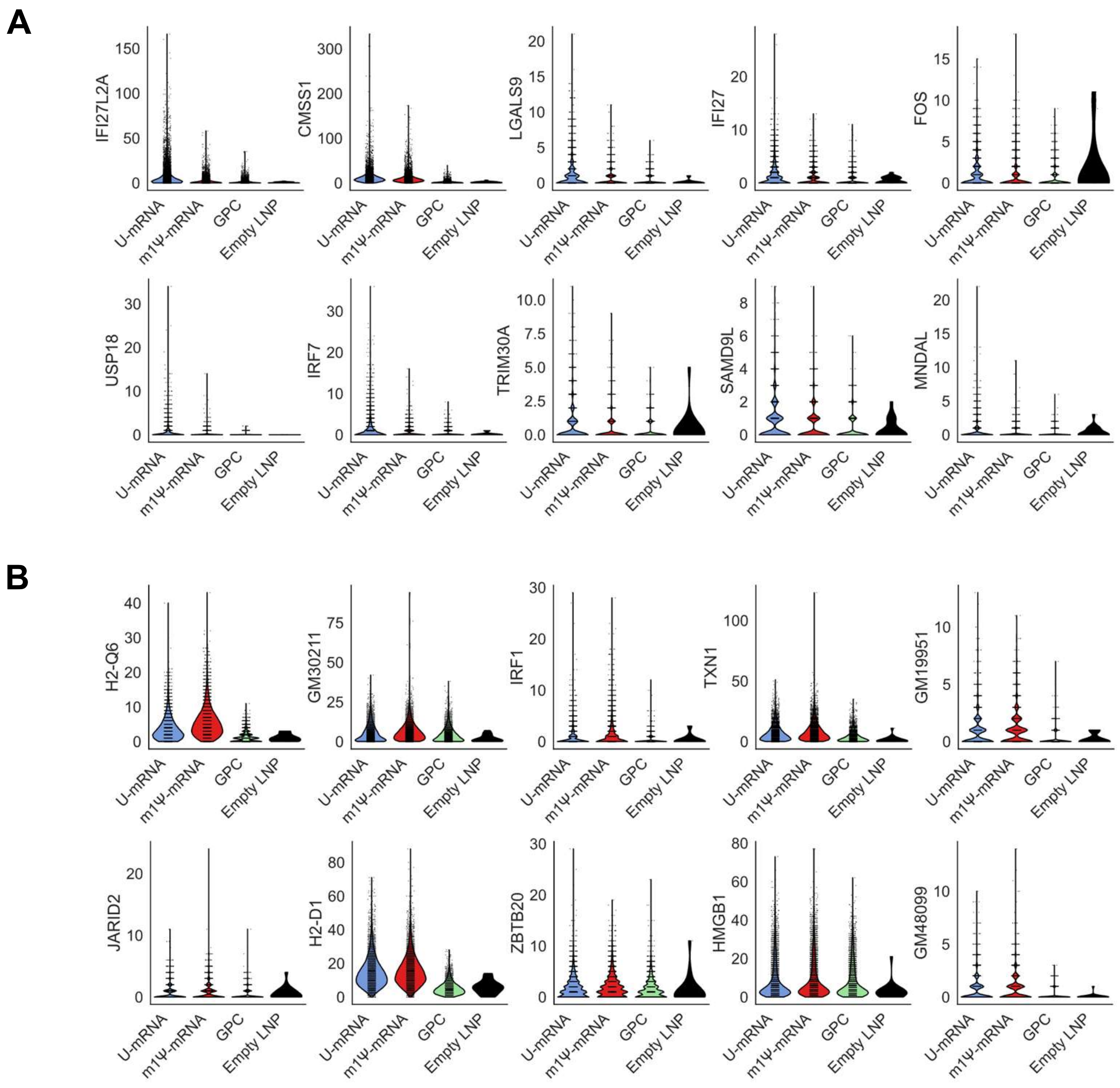

Fig. S5. Gene expression patterns in B cells of mice immunized with ANDV U-mRNA and m1Ψ-mRNA are similar. Data from the study shown in Fig. 3; n = 4 mice per group. Top 10 differentially expressed genes in B cells isolated from mice immunized with U-mRNA (**A**) and m1Ψ-mRNA (**B**).

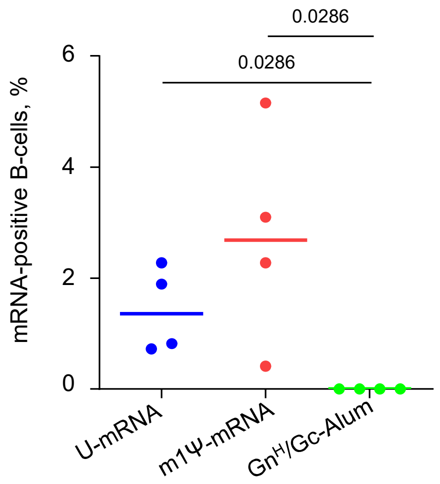

Fig. S6. Vaccine-derived U-mRNA and Ψ-mRNA persist in lymph nodes B cells of mice for at least 12 days post vaccination. Vaccine mRNA detected in the single-cell RNAseq study shown in Fig. 3; n = 4 mice per group. Proportion of cells in which vaccine mRNA was detected: values for individual mice and median values, two-tailed unpaired Mann-Whitney test.

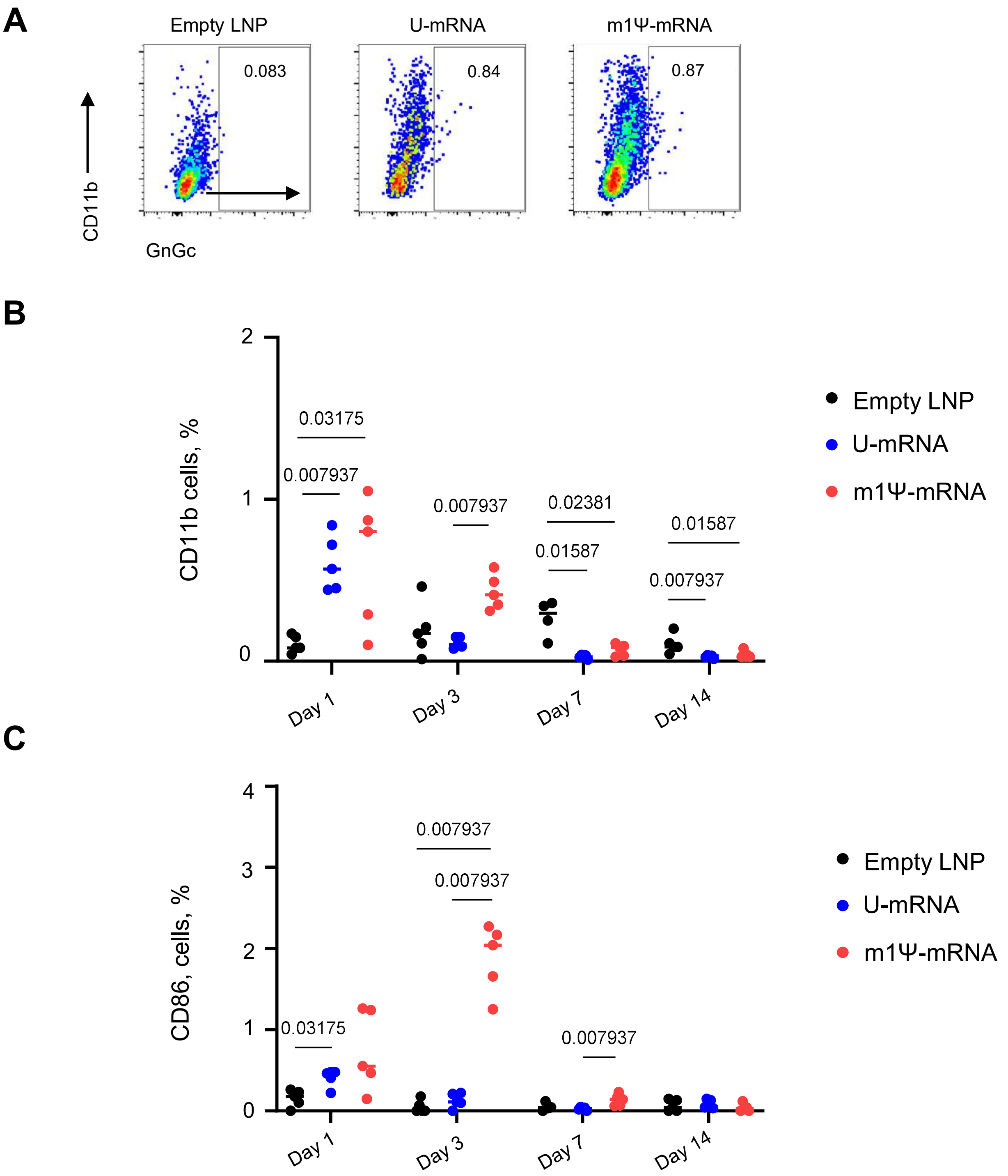

Fig. S7. Expression of the ANDV antigen by myeloid cells following vaccination. A. Representative flow cytometry plots showing presence of the GnGc antigen in CD11b population of cells, day 1 post vaccination. GnGc was gated as Lymph/Singlet/Live/B220-CD3-/CD11b+/GnGc+ (**B**) or Lymph/Singlet/Live/B220-CD3-/CD11b+/CD86+/GnGc+ (**C**) as detected by a human mAb antibody. Percentages of GnGc+ cells in CD11b+ cells (**B**) and CD11b+/CD86+ cells (**C**) from lymph nodes (pooled inguinal and popliteal) of vaccinated BALB/c mice at the indicated time points; n = 5 mice per group. Two-tailed unpaired Mann-Whitney test.

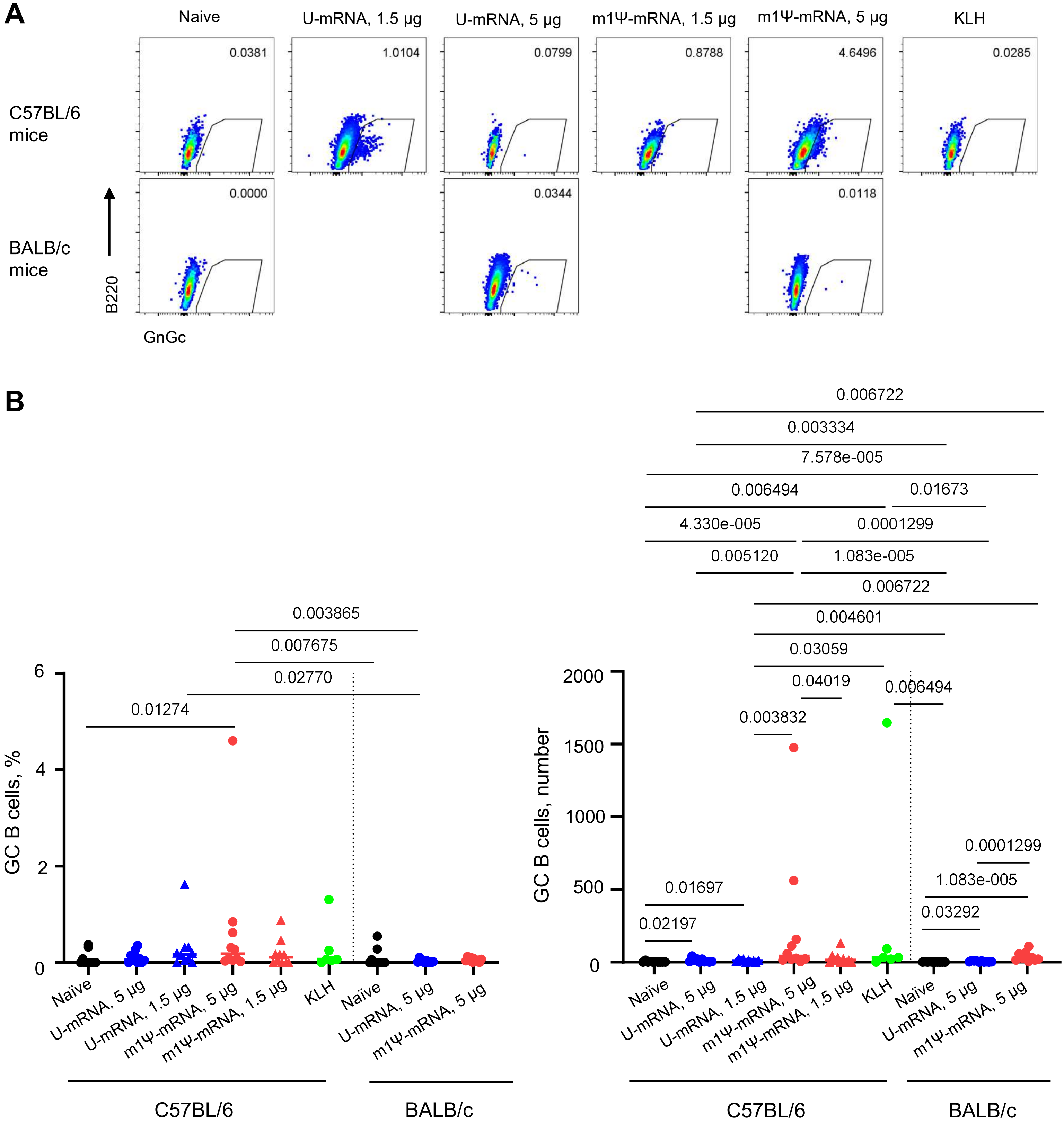

Fig. S8. Expression of the ANDV antigen by germinal center B cells following vaccination. A. A representative flow cytometry plot showing presence of the GnGc antigen. GnGc was gated as Lymph/Singlet/Live/B220+/CD4-/GL7+/CD38-/GnGc+. **B.** Percentages (B) and absolute numbers (C) of GnGc+ cells in germinal center B cells isolated from vaccinated C57BL/6J and BALB/c mice. (Naïve C57BL/6J = 10 mice, U-mRNA 5 µg C57BL/6J = 10 mice, U-mRNA 1.5 µg C57BL/6J = 10 mice, m1Ψ-mRNA 5 µg C57BL/6J = 10 mice, m1Ψ-mRNA 1.5 µg C57BL/6J = 10 mice, KLH C57BL/6J = 6 mice, Naïve BALB/c = 10 mice, U-mRNA 5 µg BALB/c = 10 mice, m1Ψ-mRNA 5 µg BALB/c = 10 mice) Two-tailed unpaired Mann-Whitney test.

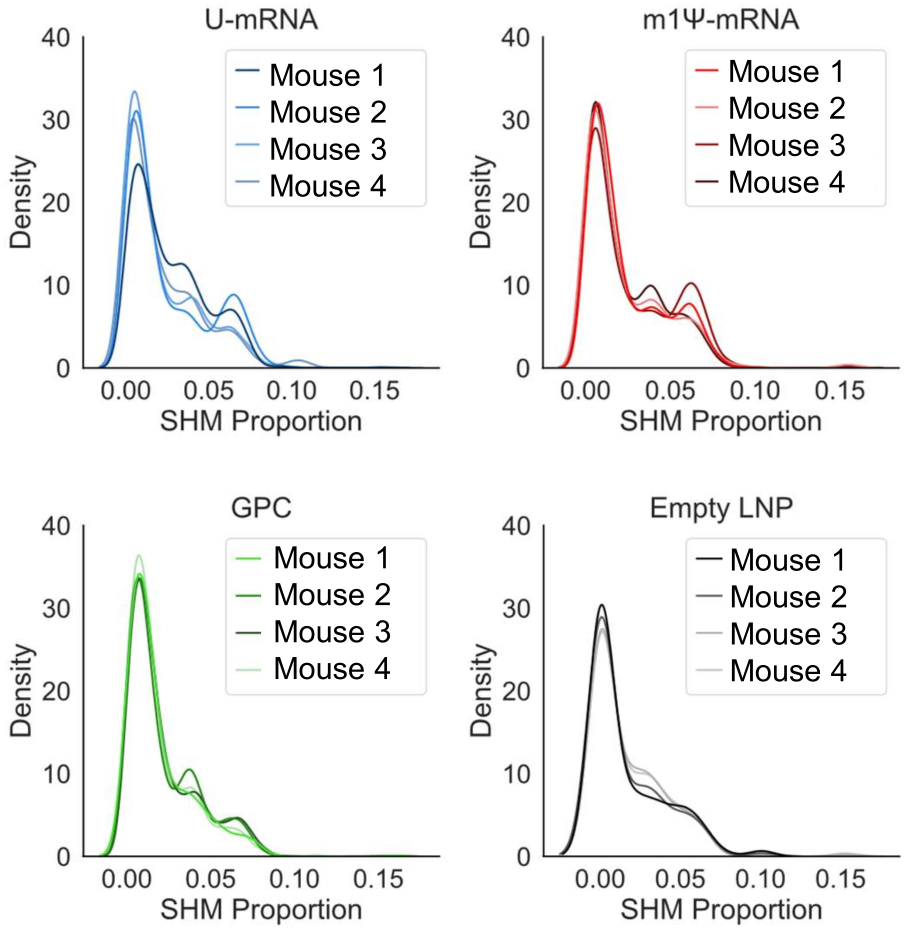

Fig. S9. Distributions of somatic hypermutations in heavy chain variable regions. Proportion of SHM is shown for the heavy chain variable region across all cells for each mouse in the experimental groups (n = 4 mice per group). Distributions are represented by a kernel density estimation.

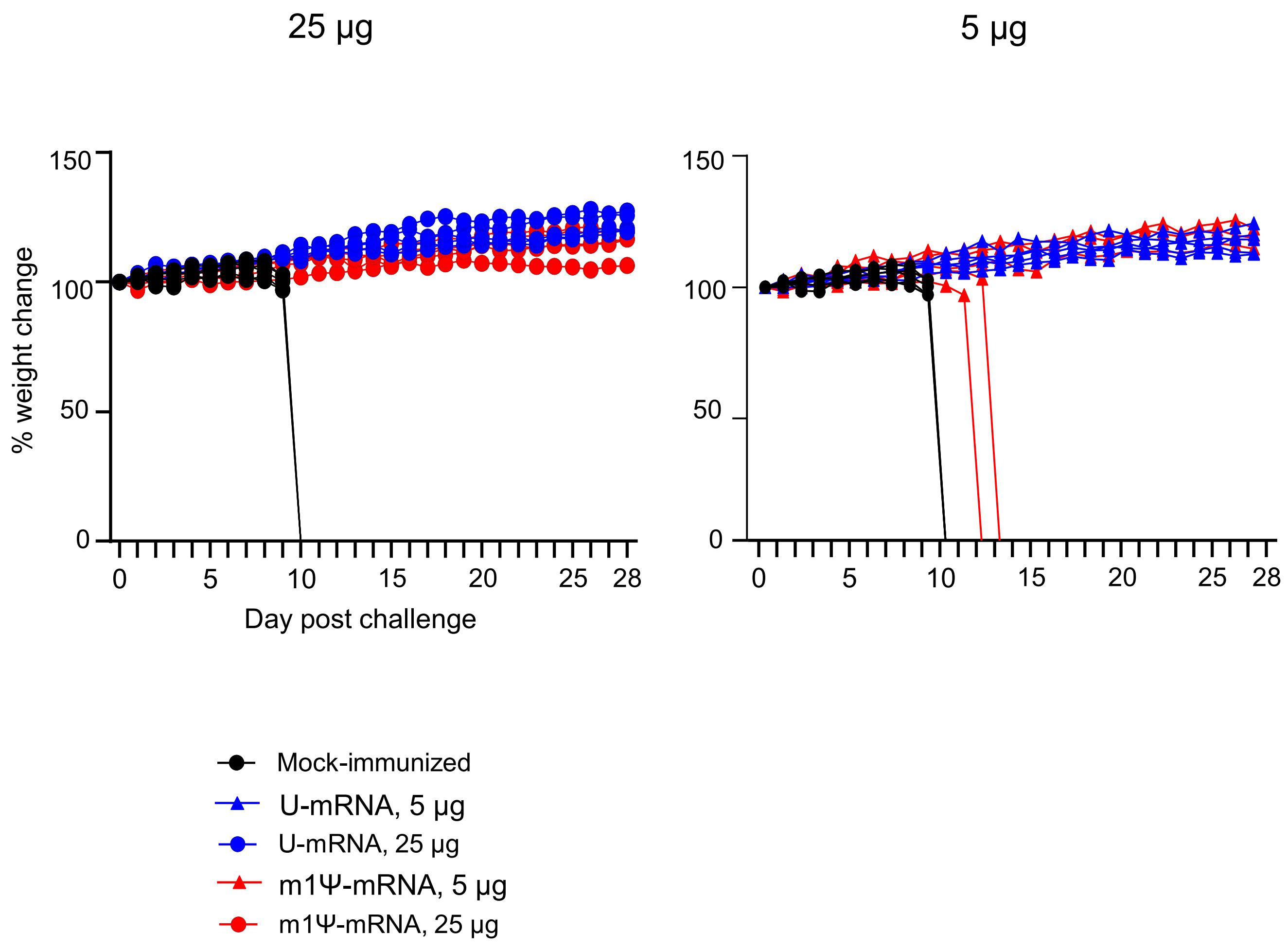

Fig. S10. Change in hamster weights in individual animals after ANDV challenge (n = 5 animals per group).

Table S1. Analysis of cytokines in A549 cells transfected with ANDV U-mRNA or m1Ψ-mRNA[1]

Target	U-mRNA	m1Ψ-mRNA
IFNA1	1.51 ± 0.5[2]	1.24 ± 0.93[2]
IFNA16	ND[3]	ND
IFNA17	ND	ND
IFNA2	ND	ND
IFNA6	ND	ND
IFNA7	ND	ND
IFNA8	ND	ND
IFNB1	17.84 ± 8.41	0.68 ± 0.83
IFNG	ND	ND
IFNL1	+[4]	ND
IFNL2	+	ND
IFNL4	+	ND
IL1A	ND	ND
IL1B	ND	ND
IL2	ND	ND
IL3	ND	ND
IL4	ND	ND
IL5	ND	ND
IL6	ND	ND
IL7	ND	ND
IL8	5.07 ± 1.1	3.33 ± 0.34
IL9	ND	ND
IL10	ND	ND
IL12A	ND	ND
IL12B	ND	ND
IL13	ND	ND
IL15	0.96 ± 0.13	0.87 ± 0.06
IL16	ND	ND
IL17A	ND	ND
IL18	1.07 ± 0.23	1.19 ± 0.21
LTA	ND	ND
TNF	ND	ND
IFIT1	208.39 ± 72.04	0.54 ± 0.15
IFIT2	133.3 ± 43.65	1.13 ± 0.27
IFIT3	94.45 ± 35.0	0.57 ± 0.20
IFIT5	5.24 ± 1.40	1.16 ± 0.05
IRF1	7.98 ± 1.88	1.29 ± 0.36
IRF9	11.78 ± 1.84	1.22 ± 0.05
MX1	578.72 ± 224.46	1.97 ± 1.09
MX2	+	ND
ISG20	8.82 ± 3.35	2.38 ± 1.01
PSMA3	1.08 ± 0.06	1.24 ± 0.14
PSMA6	1.04 ± 0.07	1.16 ± 0.02

PSME2	1.53 ± 0.25	0.99 ± 0.09
TRAFD1	1.57 ± 0.29	0.97 ± 0.08
IFIH1	37.95 ± 13.2	0.62 ± 0.10
BST2	+	ND
DDX58	30.51 ± 9.71	1.12 ± 0.02

[1] Results of qRT-PCR analysis of cytokine expression in A549 cells transfected with ANDV U-mRNA or m1Ψ-mRNA tested with TaqMan™ Array, Human Cytokine Network and custom cytokine panel (Applied Biosystems). Fold changes related mock control normalized to GAPDH are indicated (mean values ± SD based on triplicate samples).

[2] We consider the detected expression of IFNA1 mRNA non-specific or caused by the presence of remnant genomic DNA.

[3] ND, not detected.

[4] +, detected, but no quantitative comparison is possible as these genes were not detected in mock control.

Table S2.

Local expression of cytokines in muscle and draining lymph node of mice after intramuscular immunization with ANDV vaccines (10 ug), 24 h and 72 h post injection. Fold changes related to mock control, as determined by qRT-PCR using ΔΔCt method. Mean (M) and standard deviation (SD) from 3 individual animals.

Cytokine	U-mRNA								Ψ-mRNA							
	Muscle, 24 h		LN, 24 h		Muscle, 72 h		LN, 72 h		Muscle, 24 h		LN, 24 h		Muscle, 72 h		LN, 72 h	
	M	SD	M	SD	M	SD	M	SD	M	SD	M	SD	M	SD	M	SD
IFNA1	ND*	NA	ND	NA	ND	NA	ND	NA	ND	NA	ND	NA	ND	NA	ND	NA
IFNA2	ND	NA	ND	NA	ND	NA	ND	NA	ND	NA	ND	NA	ND	NA	ND	NA
IFNB	ND	NA	ND	NA	ND	NA	ND	NA	ND	NA	ND	NA	ND	NA	ND	NA
IFNG	ND	NA	ND	NA	ND	NA	ND	NA	ND	NA	ND	NA	ND	NA	ND	NA
IFNL2	ND	NA	ND	NA	ND	NA	ND	NA	ND	NA	ND	NA	ND	NA	ND	NA
CXCL10	0.27	0.09	0.99	0.87	0.19	0.02	0.71	0.58	0.88	1.16	0.67	0.50	1.04	0.52	0.64	0.28
IL15	0.49	0.11	1.27	1.28	1.12	0.14	1.19	0.25	0.59	0.74	0.96	0.50	0.63	0.46	1.44	0.52
IL16	0.72	0.34	0.57	0.34	5.28	1.81	0.99	0.25	1.80	2.48	1.86	0.94	2.69	0.97	0.98	0.37
IL18	1.09	0.50	0.32	0.43	0.17	0.06	2.54	1.79	1.04	0.53	1.10	0.59	0.12	0.03	2.40	0.82
IFIT1	0.21	0.90	0.36	0.25	0.18	0.04	0.70	0.28	0.50	0.64	0.52	0.42	1.05	0.52	0.65	0.30
IFIT3	0.14	0.08	0.58	0.25	0.29	0.11	0.72	0.32	0.79	1.23	0.47	0.33	0.11	0.06	0.63	0.28
IRF1	0.43	0.15	0.26	0.23	0.35	0.27	0.72	0.31	0.91	1.25	0.50	0.39	0.18	0.07	0.68	0.42
IRF9	0.68	0.18	0.28	0.19	1.66	0.31	1.39	0.42	2.89	3.97	1.16	0.83	0.41	0.39	0.84	0.35
MX1	1.11	0.31	0.44	0.52	1.81	0.91	0.77	0.36	3.82	5.63	1.36	1.24	0.29	0.22	1.30	0.75
MX2	1.64	0.56	0.53	0.24	0.53	0.23	0.62	0.37	2.19	2.31	0.91	0.59	0.10	0.12	0.66	0.31
ISG20	0.28	0.09	0.47	0.34	0.30	0.05	0.88	0.21	0.77	0.84	0.62	0.34	1.02	0.53	0.60	0.32
PSMA3	0.45	0.16	0.55	0.51	1.94	0.12	1.81	0.45	1.59	2.26	1.06	0.56	0.35	0.07	0.74	0.36
PSMA6	1.19	0.65	0.92	0.56	0.61	0.15	2.19	0.98	1.05	1.57	1.17	0.64	0.07	0.06	2.13	0.52
PSME2	1.09	0.52	0.66	0.85	1.08	0.55	3.24	1.02	0.36	0.57	1.33	0.69	0.18	0.11	2.67	1.20
TRAFD1	0.47	0.11	0.44	0.27	1.66	0.31	0.52	1.42	2.35	3.31	1.30	1.06	0.67	0.53	3.02	1.47
IFIH1	0.31	0.12	0.83	0.38	0.79	0.10	0.73	0.45	0.93	0.98	0.68	0.37	0.28	0.17	0.92	0.37
BST2	0.47	0.18	0.92	0.72	0.36	0.17	1.18	0.32	1.31	1.75	0.79	0.55	0.55	0.28	0.75	0.42
DDX58	0.21	0.07	0.28	0.26	0.87	0.46	0.88	0.29	1.46	1.84	0.76	0.50	0.50	0.21	1.15	0.39

* ND – not detected, NA – not applicable.

Table S3. Antibodies used in the study

Epitope	Supplier	Catalog	Clone	Lot
Flow cytometry				
B220	Biolegend	103246	RA3-6B2	B357656
CD4	Biolegend	100460	GK1.5	B376882
GL7	BD	624409	GL7	3109191
CD8	Biolegend	100766	53-6.7	B363617
CD38	Biolegend	102718	90	B352798
IgD	Biolegend	405723	11.26ac.2a	B379190
CD138	Biolegend	142518	281-2	B320884
CD86	Biolegend	105032	GL-1	B364400
CXCR4	Biolegend	146514	1.27F12	B337664
CXCR5	Biolegend	145511	L13807	B387603
PD-1	Biolegend	135219	29F.1A12	B351367
FoxP3	Biolegend	126408	MF-14	B343830
GR1	Biolegend	108423	RB6-8C5	B396926
CD11b	Biolegend	101223	M1/70	B386941
B220	Biolegend	103205	RA3-6B2	B380285
CD3	Biolegend	100205	17A2	B375691
GL7 Antigen	Biolegend	144609	GL7	B378505
CD38	Biolegend	102717	90	B385124
CD86	Biolegend	105043	GL-1	B387608
Mix-n-Stain™ CF™ 647 Antibody Labeling Kit (50-100µg) for labeling ANDV-4 Mab	Sigma Aldrich	MX647S100-1KT		22M0705K
Immunohistochemistry				
TCR-beta	Biolegend	109215	H57-597	B349876
B220	Biolegend	103240	RA3-6B2	B316684
GL7	Biolegend	144608	GL7	B255994
