# Supplementary material for: Comparison of uridine and N1-methylpseudouridine mRNA platforms in development of an Andes virus vaccine
Source: Nat Commun. 2024 Jul 30;15:6421. doi: 10.1038/s41467-024-50774-3 (PMC11289437; doi:10.1038/s41467-024-50774-3)
Supplement: Supplementary file 3 — Description of Additional Supplementary Files [file 41467_2024_50774_MOESM3_ESM.pdf]

Description of Additional Supplementary Files

File Name: Supplementary Data 1

Description: Numbers of clonal families overlapping between individual mice. Heatmap shows the number of clonal families overlapping for each of the mice, grouped by treatment. Rows and columns represent individual mice; intersections show the number of shared clonal families, with counts colored as heat. The diagonal (colored grey) contains self-comparisons representing the total number of clonal families within a mouse.

File Name: Supplementary Data 2

Description: Filtering metrics for Scanpy and Cell Ranger analysis of sequencing outputs.

Contains the following sheets:

Filtering: Filters applied and resulting counts for each sample in Scanpy.

VDJ CellRanger: Quality control metrics for sequences provided by CellRanger VDJ pipeline.

GEX CellRanger: Quality control metrics for sequences provided by CellRanger counts pipeline.
