# Supplementary material for: Comparison of uridine and N1-methylpseudouridine mRNA platforms in development of an Andes virus vaccine
Source: Nat Commun. 2024 Jul 30;15:6421. doi: 10.1038/s41467-024-50774-3 (PMC11289437; doi:10.1038/s41467-024-50774-3)
Supplement: Supplementary file 6 — Reporting Summary [file 41467_2024_50774_MOESM6_ESM.pdf]

nature portfolio

Corresponding author(s): *Bukreyev, Alexander*

Last updated by author(s): *07/16/2024*

Reporting Summary

Nature Portfolio wishes to improve the reproducibility of the work that we publish. This form provides structure for consistency and transparency in reporting. For further information on Nature Portfolio policies, see our Editorial Policies and the Editorial Policy Checklist.

Statistics

For all statistical analyses, confirm that the following items are present in the figure legend, table legend, main text, or Methods section.

n/a	Confirmed	
☐	☒	The exact sample size (*n*) for each experimental group/condition, given as a discrete number and unit of measurement
☒	☐	A statement on whether measurements were taken from distinct samples or whether the same sample was measured repeatedly
☐	☒	The statistical test(s) used AND whether they are one- or two-sided<br>*Only common tests should be described solely by name; describe more complex techniques in the Methods section.*
☐	☒	A description of all covariates tested
☐	☒	A description of any assumptions or corrections, such as tests of normality and adjustment for multiple comparisons
☐	☒	A full description of the statistical parameters including central tendency (e.g. means) or other basic estimates (e.g. regression coefficient) AND variation (e.g. standard deviation) or associated estimates of uncertainty (e.g. confidence intervals)
☐	☒	For null hypothesis testing, the test statistic (e.g. *F*, *t*, *r*) with confidence intervals, effect sizes, degrees of freedom and *P* value noted<br>*Give P values as exact values whenever suitable.*
☒	☐	For Bayesian analysis, information on the choice of priors and Markov chain Monte Carlo settings
☒	☐	For hierarchical and complex designs, identification of the appropriate level for tests and full reporting of outcomes
☒	☐	Estimates of effect sizes (e.g. Cohen's *d*, Pearson's *r*), indicating how they were calculated

Our web collection on statistics for biologists contains articles on many of the points above.

Software and code

Policy information about availability of computer code

Data collection	*Illumina software; BD Accuri C6 Plus; BD LSR Fortessa software; BD FACSaria Fusion sorter*
Data analysis	*GraphPad Prism v. 9.5; SCANPY; GSEAPY; IMGT/High-V Quest; Flow Jo v. 10. Scripts for analysis of the single-cell RNA-seq and BCR-seq data are available as Jupyter notebooks at https://github.com/IGlab-VUMC/Andes_Virus_SC-analysis.*<br>*Scripts for analysis of the single-cell RNA-seq and BCR-seq data are available as Jupyter notebooks at https://github.com/IGlab-VUMC/Andes_Virus_SC-analysis.*

For manuscripts utilizing custom algorithms or software that are central to the research but not yet described in published literature, software must be made available to editors and reviewers. We strongly encourage code deposition in a community repository (e.g. GitHub). See the Nature Portfolio guidelines for submitting code & software for further information.

Data

Policy information about availability of data

All manuscripts must include a data availability statement. This statement should provide the following information, where applicable:

- Accession codes, unique identifiers, or web links for publicly available datasets
- A description of any restrictions on data availability
- For clinical datasets or third party data, please ensure that the statement adheres to our policy

The mRNA vaccine construct sequence has been deposited in GenBank under accession number PP784251 [https://www.ncbi.nlm.nih.gov/nuccore/?

term=PP784251]. Reference sequence of ANDV segment M used for the development of mRNA is available in GenBank under accession code NC_003467 [https://www.ncbi.nlm.nih.gov/nuccore/NC_003467]; the same virus was used in our in-vitro and in-vivo studies. Sequence of SNV used in the in-vitro studies is available under accession number AF281850 [https://www.ncbi.nlm.nih.gov/nuccore/AF281850]. Single cell sequencing data is available via the Gene Expression Omnibus database (GEO) under accession code GSE240064 [https://www.ncbi.nlm.nih.gov/geo/query/acc.cgi?acc=GSE240064]. Data used for graphs are available in the Source Data file. All other data is available from authors upon request.

Research involving human participants, their data, or biological material

Policy information about studies with human participants or human data. See also policy information about sex, gender (identity/presentation), and sexual orientation and race, ethnicity and racism.

Reporting on sex and gender	Not applicable
Reporting on race, ethnicity, or other socially relevant groupings	Not applicable
Population characteristics	Not applicable
Recruitment	Not applicable
Ethics oversight	Not applicable

Note that full information on the approval of the study protocol must also be provided in the manuscript.

Field-specific reporting

Please select the one below that is the best fit for your research. If you are not sure, read the appropriate sections before making your selection.

[x] Life sciences [ ] Behavioural & social sciences [ ] Ecological, evolutionary & environmental sciences

For a reference copy of the document with all sections, see nature.com/documents/nr-reporting-summary-flat.pdf

Life sciences study design

All studies must disclose on these points even when the disclosure is negative.

Sample size	For in-vitro studies, biological replicates were used as the minimum suitable for statistical comparisons. Deviations in these triplicates were limited and statistical support significant.<br>Syrian hamsters demonstrate 100% mortality when infected with high doses of ANDV. This is what we expect in our control group. However, vaccine efficiency may vary, particularly depending on dose and regimen of administration. Group sample sizes of 5 in experimental and control groups achieve 83% power to detect a difference between the group proportions of 0.849. The proportion in the treatment group is assumed to be 0.001 under the null hypothesis and 0.85 under the alternative hypothesis. The proportion in the control group is 0.001. The test statistic used is the two-sided Fisher's Exact Test. The significance level of the test is targeted at 0.05.<br>For the mouse study of GC development, group sample sizes of 9 achieve 89.29% power to reject the null hypothesis of equal means when the population mean difference is $\mu1 - \mu2 = 1.6 - 0 = 1.6$ with a standard deviation for both groups of 1 and with a significance level (alpha) of 0.05 using a two-sided equal-variance t-test.<br>For B-cell repertoire sequencing we used mice with group size of 4, mainly because of very high costs of this study. At 95% confidence level (2-tailed unpaired T-test) a sample size of 4 animals per group at each time point will provide greater than 80% power to detect an increase in mean of the log transformed measure of 2 assuming a standard deviation of 0.8.
Data exclusions	No data were excluded.
Replication	All experiments were done in biological triplicates as the minimum, or on groups of 4 to 18 animals. Some of the animal studies were done as 2-3 independent identical experiments (to limit animal number per each experiment to make it doable). Electron microscopy experiment was performed twice. The details are provided in figure captions. All results were reproducible, as also demonstrated by statistical analysis.
Randomization	The animals were randomly assigned to experimental groups.
Blinding	No blinding was implemented.

Behavioural & social sciences study design

All studies must disclose on these points even when the disclosure is negative.

Study description	Not applicable.
Research sample	Not applicable.
Sampling strategy	Not applicable.

Data collection	*Not applicable.*
Timing	*Not applicable.*
Data exclusions	*Not applicable.*
Non-participation	*Not applicable.*
Randomization	*Not applicable.*

Ecological, evolutionary & environmental sciences study design

All studies must disclose on these points even when the disclosure is negative.

Study description	*Not applicable.*
Research sample	*Not applicable.*
Sampling strategy	*Not applicable.*
Data collection	*Not applicable.*
Timing and spatial scale	*Not applicable.*
Data exclusions	*Not applicable.*
Reproducibility	*Not applicable.*
Randomization	*Not applicable.*
Blinding	*Not applicable.*

Did the study involve field work? ☐ Yes ☒ No

Field work, collection and transport

Field conditions	*Not applicable.*
Location	*Not applicable.*
Access & import/export	*Not applicable.*
Disturbance	*Not applicable.*

Reporting for specific materials, systems and methods

We require information from authors about some types of materials, experimental systems and methods used in many studies. Here, indicate whether each material, system or method listed is relevant to your study. If you are not sure if a list item applies to your research, read the appropriate section before selecting a response.

Materials & experimental systems

n/a	Involved in the study	
☐	☒	Antibodies
☐	☒	Eukaryotic cell lines
☒	☐	Palaeontology and archaeology
☐	☒	Animals and other organisms
☒	☐	Clinical data
☒	☐	Dual use research of concern
☒	☐	Plants

Methods

n/a	Involved in the study	
☒	☐	ChIP-seq
☐	☒	Flow cytometry
☒	☐	MRI-based neuroimaging

Antibodies

Antibodies used	*Experimental antibodies ANDV-4, -5, -12, -22, -23, -34, and -44 targeting ANDV Gn/Gc, were used to detect expression of ANDV glycoprotein by flow cytometry. These were described previously, doi: 10.1016/j.celrep.2021.109453. Anti-mouse antibodies used for cell sorting prior to the GC analysis and single cell sequencing were sourced from Biolegend and Beckton Dickenson. These included: 11-26c.a2 (IgD), 281-2 (CD138), RA3-6B2 (B220), GL7 (GL7), 90 (CD38), GL-1 (CD86), 1.27F12 (CXCR4), GK1.5 (CD4), 53-6.7 (CD8), LI38D7 (CXCR5), 29F.1A12 (PD-1), and MF-14 (FoxP3). Details on antibodies are present in Table S3.*
Validation	*The ANDV antibodies were described previously, doi: 10.1016/j.celrep.2021.109453. In our assays, the only validation was staining of mock-transfected cells with these antibodies. No validation was performed for the commercially available anti-mouse antibodies used for cell sorting prior to GC evaluation and single cell sequencing except that beads with corresponding fluorofores were used for laser signal adjustments.*

Eukaryotic cell lines

Policy information about cell lines and Sex and Gender in Research

Cell line source(s)	*Vero E6 (ATCC #CRL-1586), A549 (ATCC # CCL-185), 293T (ATCC # CRL-3216).*
Authentication	*None of the cell lines used were authenticated.*
Mycoplasma contamination	*All cell lines tested negative for mycoplasma contamination*
Commonly misidentified lines (See ICLAC register)	*Not applicable.*

Palaeontology and Archaeology

Specimen provenance	*Not applicable.*
Specimen deposition	*Not applicable.*
Dating methods	*Not applicable.*

☐ Tick this box to confirm that the raw and calibrated dates are available in the paper or in Supplementary Information.

Ethics oversight	*Not applicable.*

Note that full information on the approval of the study protocol must also be provided in the manuscript.

Animals and other research organisms

Policy information about studies involving animals; ARRIVE guidelines recommended for reporting animal research, and Sex and Gender in Research

Laboratory animals	*Mice C57Bl6/J (10-12 weeks-old; The Jackson Laboratory), BALB/c (10 weeks-old; The Jackson Laboratory), golden Syrian hamsters (10 weeks-old; Charles River)*
Wild animals	*Not applicable.*
Reporting on sex	*Females only were used to avoid unwanted breeding during the experiment and excessive fighting of males. For the sample size used, no sex-related differences were anticipated.*
Field-collected samples	*Not applicable.*
Ethics oversight	*UTMB IACUC*

Note that full information on the approval of the study protocol must also be provided in the manuscript.

Clinical data

Policy information about clinical studies

All manuscripts should comply with the ICMJEguidelines for publication of clinical research and a completedCONSORT checklist must be included with all submissions.

Clinical trial registration	*Not applicable.*

Study protocol	*Not applicable.*
Data collection	*Not applicable.*
Outcomes	*Not applicable.*

Dual use research of concern

Policy information about dual use research of concern

Hazards

Could the accidental, deliberate or reckless misuse of agents or technologies generated in the work, or the application of information presented in the manuscript, pose a threat to:

No	Yes	
☒	☐	Public health
☒	☐	National security
☒	☐	Crops and/or livestock
☒	☐	Ecosystems
☒	☐	Any other significant area

Experiments of concern

Does the work involve any of these experiments of concern:

No	Yes	
☒	☐	Demonstrate how to render a vaccine ineffective
☒	☐	Confer resistance to therapeutically useful antibiotics or antiviral agents
☒	☐	Enhance the virulence of a pathogen or render a nonpathogen virulent
☒	☐	Increase transmissibility of a pathogen
☒	☐	Alter the host range of a pathogen
☒	☐	Enable evasion of diagnostic/detection modalities
☒	☐	Enable the weaponization of a biological agent or toxin
☒	☐	Any other potentially harmful combination of experiments and agents

Plants

Seed stocks	*Not applicable.*
Novel plant genotypes	*Not applicable.*
Authentication	*Not applicable.*

ChIP-seq

Data deposition

☐ Confirm that both raw and final processed data have been deposited in a public database such as GEO.

☐ Confirm that you have deposited or provided access to graph files (e.g. BED files) for the called peaks.

Data access links<br>*May remain private before publication.*	*Not applicable.*
Files in database submission	*Not applicable.*
Genome browser session<br>(e.g. UCSC)	*Not applicable.*

Methodology

Replicates	*Not applicable.*

Sequencing depth	*Not applicable.*
Antibodies	*Not applicable.*
Peak calling parameters	*Not applicable.*
Data quality	*Not applicable.*
Software	*Not applicable.*

Flow Cytometry

Plots

Confirm that:

- [ ] The axis labels state the marker and fluorochrome used (e.g. CD4-FITC).
- [x] The axis scales are clearly visible. Include numbers along axes only for bottom left plot of group (a 'group' is an analysis of identical markers).
- [ ] All plots are contour plots with outliers or pseudocolor plots.
- [x] A numerical value for number of cells or percentage (with statistics) is provided.

Methodology

Sample preparation	*1. In-vitro evaluation of ANDV glycoprotein expression. Cells transfected with ANDV U-mRNA or m1Ψ-mRNA in 24-well plate format were digested in 0.25 ml of 0.25% trypsin-EDTA (Gibco) which was further neutralized by an addition of 0.25 ml of calf serum (Corning). Cells were washed by PBS, fixed in 4% paraformaldehyde (Polysciences, Inc), and subjected to immunostaining with a cocktail of human monoclonal antibodies ANDV-4, -5, -12, -22, -23, -34, and -44 targeting ANDV Gn/Gc and capable to both bind and neutralize the virus 10 diluted to 2 μg/ml in StartingBlock T 20 (TBS) Blocking Buffer (Thermo Scientific). After incubation during 1 h at room temperature and two PBS washes, cells were incubated with the secondary goat anti-human FITC-conjugated antibody (Southern Biotech) diluted 1:500 in TBS and incubated at the same conditions in the dark. Stained cells were double-washed in PBS and subjected to flow cytometry on Accuri C6.*<br>*2. GC analysis: Cells from inguinal and popliteal lymph nodes were pooled for each mouse. Cells were counted, Fc blocked, and stained with indicated monoclonal antibodies that were commercially sourced (Biolegend and Beckton Dickenson). Key antibody clones used were: 11-26c.a2 (IgD), 281-2 (CD138), RA3-6B2 (B220), GL7 (GL7), 90 (CD38), GK1.5 (CD4), 53-6.7 (CD8). Cells were acquired on a BD LSR Fortessa.*
Instrument	*BD Accuri C6; BD LSR Fortessa.*
Software	*BD Accuri C6 Plus; BD LSR Fortessa; Flow Jo v.10.*
Cell population abundance	*Relative frequencies of GC populations are reported (e.g. of total live B cells). Absolute counts of live lymphocytes for each sample were manually determined, enabling total cell numbers to be calculated. For cell sorting experiments, due to limited sample available post sort and the 10x genomics workflow a direct purity check was not feasible. The cell sorter was quality control checked using CST and Accudrop beads and was shown to be highly stable. The sequencing data confirmed that the sorted cells were indeed GC B cells by upregulation of prototypical GC B cell genes (e.g. aicda).*
Gating strategy	*Germinal center (GC) B cells were gated using well established conventional markers that are expressed by GC B cells.*<br>*The population was obvious and had clear separation from non-GC B cells. GC B cells were gated as single live lymphocytes (FSC-A vs SSC-A and SSC-A vs H) that are B220+, CD4-, CD8-, GL7+, CD38-, IgD , CD138- and purified by cell sorting using the BD FACSAria Fusion sorter (Beckton Dickinson, Inc).*

- [ ] Tick this box to confirm that a figure exemplifying the gating strategy is provided in the Supplementary Information.

Magnetic resonance imaging

Experimental design

Design type	*Indicate task or resting state; event-related or block design.*
Design specifications	*Specify the number of blocks, trials or experimental units per session and/or subject, and specify the length of each trial or block (if trials are blocked) and interval between trials.*
Behavioral performance measures	*State number and/or type of variables recorded (e.g. correct button press, response time) and what statistics were used to establish that the subjects were performing the task as expected (e.g. mean, range, and/or standard deviation across subjects).*

Acquisition

Imaging type(s) — *Specify: functional, structural, diffusion, perfusion.*

Field strength — *Specify in Tesla*

Sequence & imaging parameters — *Specify the pulse sequence type (gradient echo, spin echo, etc.), imaging type (EPI, spiral, etc.), field of view, matrix size, slice thickness, orientation and TE/TR/flip angle.*

Area of acquisition — *State whether a whole brain scan was used OR define the area of acquisition, describing how the region was determined.*

Diffusion MRI ☐ Used ☐ Not used

Preprocessing

Preprocessing software — *Provide detail on software version and revision number and on specific parameters (model/functions, brain extraction, segmentation, smoothing kernel size, etc.).*

Normalization — *If data were normalized/standardized, describe the approach(es): specify linear or non-linear and define image types used for transformation OR indicate that data were not normalized and explain rationale for lack of normalization.*

Normalization template — *Describe the template used for normalization/transformation, specifying subject space or group standardized space (e.g. original Talairach, MNI305, ICBM152) OR indicate that the data were not normalized.*

Noise and artifact removal — *Describe your procedure(s) for artifact and structured noise removal, specifying motion parameters, tissue signals and physiological signals (heart rate, respiration).*

Volume censoring — *Define your software and/or method and criteria for volume censoring, and state the extent of such censoring.*

Statistical modeling & inference

Model type and settings — *Specify type (mass univariate, multivariate, RSA, predictive, etc.) and describe essential details of the model at the first and second levels (e.g. fixed, random or mixed effects; drift or auto-correlation).*

Effect(s) tested — *Define precise effect in terms of the task or stimulus conditions instead of psychological concepts and indicate whether ANOVA or factorial designs were used.*

Specify type of analysis: ☐ Whole brain ☐ ROI-based ☐ Both

Statistic type for inference — *Specify voxel-wise or cluster-wise and report all relevant parameters for cluster-wise methods.*

(See Eklund et al. 2016)

Correction — *Describe the type of correction and how it is obtained for multiple comparisons (e.g. FWE, FDR, permutation or Monte Carlo).*

Models & analysis

n/a	Involved in the study
☐	☐ Functional and/or effective connectivity
☐	☐ Graph analysis
☐	☐ Multivariate modeling or predictive analysis

Functional and/or effective connectivity — *Report the measures of dependence used and the model details (e.g. Pearson correlation, partial correlation, mutual information).*

Graph analysis — *Report the dependent variable and connectivity measure, specifying weighted graph or binarized graph, subject- or group-level, and the global and/or node summaries used (e.g. clustering coefficient, efficiency, etc.).*

Multivariate modeling and predictive analysis — *Specify independent variables, features extraction and dimension reduction, model, training and evaluation metrics.*
